# Cloning the barley *nec3* disease lesion mimic mutant using complementation by sequencing

**DOI:** 10.1002/tpg2.20187

**Published:** 2022-03-18

**Authors:** Serena Rosignoli, Francesco Cosenza, Matthew J. Moscou, Laura Civolani, Francesco Musiani, Cristian Forestan, Sara Giulia Milner, Castrense Savojardo, Roberto Tuberosa, Silvio Salvi

**Affiliations:** ^1^ Dep. of Agricultural and Food Sciences Univ. of Bologna Viale G. Fanin 44 Bologna, Italy 40127; ^2^ The Sainsbury Laboratory Univ. of East Anglia Norwich Research Park Norwich NR4 7UK UK; ^3^ Laboratory of Bioinorganic Chemistry, Dep. of Pharmacy and Biotechnology Univ. of Bologna Via Belmeloro 6 Bologna, Italy 40126; ^4^ Leibniz Institute of Plant Genetics and Crop Plant Research (IPK) Gatersleben, Seeland, D; ^5^ Biocomputing Group, Dep. of Pharmacy and Biotechnology Univ. of Bologna Via Belmeloro 6 Bologna, Italy 40126

## Abstract

Disease lesion mimic (DLM) or necrotic mutants display necrotic lesions in the absence of pathogen infections. They can show improved resistance to some pathogens and their molecular dissection can contribute to revealing components of plant defense pathways. Although forward‐genetics strategies to find genes causal to mutant phenotypes are available in crops, these strategies require the production of experimental cross populations, mutagenesis, or gene editing and are time‐ and resource‐consuming or may have to deal with regulated plant materials. In this study, we described a collection of 34 DLM mutants in barley (*Hordeum vulgare* L.) and applied a novel method called complementation by sequencing (CBS), which enables the identification of the gene responsible for a mutant phenotype given the availability of two or more chemically mutagenized individuals showing the same phenotype. Complementation by sequencing relies on the feasibility to obtain all induced mutations present in chemical mutants and on the low probability that different individuals share the same mutated genes. By CBS, we identified a *cytochrome P450 CYP71P1* gene as responsible for orange blotch DLM mutants, including the historical barley *nec3* locus. By comparative phylogenetic analysis we showed that *CYP71P1* gene family emerged early in angiosperm evolution but has been recurrently lost in some lineages including *Arabidopsis thaliana* (L.) Heynh. Complementation by sequencing is a straightforward cost‐effective approach to clone genes controlling phenotypes in a chemically mutagenized collection. The TILLMore (TM) collection will be instrumental for understanding the molecular basis of DLM phenotypes and to contribute knowledge about mechanisms of host–pathogen interaction.

## Core Ideas

- Disease lesion mimic (DLM) mutants are instrumental in studying host–pathogen interaction.
- A collection of 34 new disease lesion mimic mutants in barley is described.
- The cost‐effective gene cloning method complementation by sequencing is introduced.
- *Cytochrome P450 CYP71P1* was found responsible for the historical *nec3* DLM barley mutant.
- *Cytochrome P450 CYP71P1* gene family emerged early in plant evolution and was lost in *Arabidopsis*.

**Abbreviations**
BSA: bulk segregant analysis
CBS: complementation by sequencing
DLM: disease lesion mimic
EMS: ethyl methanesulfonate
KASP: Kompetitive allele specific polymerase chain reaction
MGD: mutant gene density
SNP: single‐nucleotide polymorphism
T5H: tryptamine 5‐hydroxylase
TDC: tryptophan decarboxylase
TM: barley chemically mutagenized TILLMore population
WGS: whole‐genome shotgun.

## 1 INTRODUCTION

Collections of artificially (chemically or physically) random‐induced mutants have proved extremely valuable for basic biology investigations as well as for crop improvement and varietal release (Lundqvist, 2014; Mba, 2013; Oladosu et al., 2016; Parry et al., 2009). Disease lesion mimic (DLM) mutants are a heterogenous class of mutants showing colored spots, necrosis, or other types of spontaneous lesions, mainly on leaf blades, similar to disease‐related phenotypes (Balint‐Kurti, 2019; Bruggeman et al., 2015). Tens of DLM mutants have been identified in barley, maize (*Zea mays* L.), rice (*Oryza sativa* L.), *Arabidopsis* (Bruggeman et al., 2015; Zheng et al., 2021), and occasionally in other species including groundnut (*Arachis hypogaea* L.) (Badigannavar et al., 2002), soybean [*Glycine max* (L.) Merr.] (Al Amin et al., 2019), tomato (*Solanum lycopersicum* L.) (Spassieva & Hille, 2002), and switchgrass (*Panicum virgatum* L.) (Liu et al., 2017). In many cases, the DLM phenotype can be described as a form of the well‐known hypersensitive response characterized by a rapid and localized cell death. Thus, DLM mutants usually suffer negative pleiotropic effects including reduced photosynthesis, retarded growth, and increased susceptibility to necrotrophic pathogens (Balint‐Kurti, 2019). This notwithstanding, DLM‐causing alleles were occasionally shown to contribute to resistance to pathogens, as demonstrated for the barley *mlo* and *nec1* recessive genes for powdery mildew (*Blumeria graminis* f. sp. *hordei*) and bacterial pathogens, respectively (Büschges et al., 1997; Keisa et al., 2011; Kumar et al., 2001). Similarly, DLM *Spl17* and *Spl26* confer resistance to *Magnaporthe oryzae* and *Xanthomonas oryzae* in rice (Wu et al., 2008), *lm3* confers powdery mildew (*Blumeria gramini*s f. sp. *tritici*) resistance in wheat (*Triticum aestivum* L.) (Wang et al., 2016), *Lm5* enhances resistance to powdery mildew and stripe rust (*Puccinia striiformis* f.sp. *tritici*) in wheat (Li et al., 2021), and *Rp1‐D21* confers resistance to rust (*Puccinia sorghi*) in maize (Smith et al., 2010). In wheat, the complementary genes *Necrosis 1* (*Ne1*) and *Necrosis 2* (*Ne2*) cause hybrid necrosis (Chu et al., 2006; Tsunewaki, 1970), a type of hybrid postzygotic incompatibility associated with necrosis on the leaves of F_1_ seedlings, stunted growth and sometimes lethality caused by deleterious epistatic interaction with a range of symptoms connected to multiple alleles at both loci. The *Ne2* gene was recently cloned and demonstrated to be the same gene as *Lr13* (Hewitt et al., 2021; Si et al., 2021), the widely distributed resistance gene against leaf rust caused by *Puccinia triticina*.

**Core Ideas**

- Disease lesion mimic (DLM) mutants are instrumental in studying host–pathogen interaction.
- A collection of 34 new disease lesion mimic mutants in barley is described.
- The cost‐effective gene cloning method complementation by sequencing is introduced.
- *Cytochrome P450 CYP71P1* was found responsible for the historical *nec3* DLM barley mutant.
- *Cytochrome P450 CYP71P1* gene family emerged early in plant evolution and was lost in *Arabidopsis*.

Cloning genes responsible for chemically or physically induced mutations showing simple Mendelian inheritance has been carried out using positional cloning approaches, which have been recently complemented by mapping‐by‐sequencing and its different implementations (Bettgenhaeuser & Krattinger, 2019; Schneeberger, 2014). Cloning a gene by mapping‐by‐sequencing relies on (a) production of a mapping population (e.g., an F_2_, from a cross between two inbred lines, one carrying the target yet unknown mutation), (b) bulk segregant analysis (BSA) (Michelmore et al., 1991) using next‐generation sequencing approaches for high‐density single‐nucleotide polymorphism (SNP) genotyping, and (c) identification of SNPs with significantly altered allele frequencies between wild‐type and mutant DNA bulks. Ideally, mapping‐by‐sequencing is expected to highlight the causative SNP, leading to gene cloning. If this is not the case, SNPs genetically linked to the causative gene are, however, expected to be identified. Although mapping‐by‐sequencing was very successful in both model and crop species (Mascher et al., 2014; Schneeberger, 2014), it still requires the production of experimental cross‐populations and genetic mapping information, which could be quite demanding for some species. Attempts to circumvent this bottleneck, that is, cloning by sequencing without mapping, have been recognized as possible if alternative methods exist to confirm the target gene function in the species (Candela et al., 2015). This approach was first applied in fruit fly (*Drosophila melanogaster*), where a gene controlling egg‐shell morphology was identified by whole‐genome shotgun (WGS) sequencing of independent ethyl methanesulfonate (EMS)‐induced mutants showing the same egg phenotype followed by confirmation by complementation (Blumenstiel et al., 2009). Similar experiments were carried out in other model species, for example, in nematode (*Caenorhabditis elegans*) (Sarin et al., 2008). Despite these promising results, this approach has yet to be fully extended to plants, although some attempts were made. For example, in barley and wheat, a combination of WGS sequencing and chromosome flow sorting, named ‘MutChromSeq,’ was applied to multiple mutants to clone the underlying gene, provided that the chromosome carrying the target gene was known (Sánchez‐Martín et al., 2016). An optimized combination of next‐generation sequencing for induced SNP discovery and progeny testing enabled Heuermann et al. (2019) to clone maize mutant genes, but this still required the production of controlled experimental crosses followed by genome‐sequencing 16 plants. In *Arabidopsis*, a protocol named ‘mutagenomics’ was recently developed that combined EMS mutagenesis, phenotypic screening, WGS sequencing, and progeny testing (Hodgens et al., 2020).

Here we describe a novel collection of DLM mutants in barley and develop a phenotype‐driven, WGS sequencing‐based approach, which we named ‘complementation by sequencing’ (CBS), suitable for identifying candidate genes for target mutations without using genetic information, experimental cross‐populations, or progeny testing. We applied this method to clone the gene responsible for three independent orange‐blotch DLM mutations, and we showed that this gene corresponds to the historical barley *nec3* mutant (Lundqvist et al., 1997).

## 2 MATERIALS AND METHODS

### 2.1 Plant material and growth conditions

All the barley necrotic mutants hereby referred to, except for BW630, belong to the TILLMore (TM) collection of barley obtained with sodium azide mutagenesis on the cultivar Morex (Talamè et al., 2008). Two F_2_ mapping populations were generated from the cross TM599 × Barke and TM4118 × Barke. The F_1_ population from the cross TM599 × BW630, and the F_1_ population from TM185 × TM599 and TM599 × TM1000 were created to perform complementation tests. BW630 is a Bowman backcross‐derived line (Druka et al., 2011) carrying an orange blotch DLM *nec3* allele originally generated by X‐ray mutagenesis on the cultivar Villa (Häuser & Fischbeck, 1976). BW630 seeds were kindly supplied by Luke Ramsay from the James Hutton Institute, Invergowrie, UK, and are deposited at the Nordic Genetic Resource Center, Sweden. In order to morphologically classify the DLM mutants, necrosis was observed in terms of their approximate shape, margin (distinct or blurred), dimension (small, main axis < 3 mm; medium, main axis between 3 and 6 mm, inclusive; large, main axis > 6 mm), and color.

The entire TM collection, and the cultivars Morex and Barke, were grown in open field in Cadriano (44°33′03’’ N, 11°24′36’’ E; 33 m asl), Italy, in the spring of 2016 and again in the spring of 2019 following standard agronomic practices in 0.6‐m‐long two‐row plots with a randomized design. The two F_2_ populations and their parental lines were grown in the open field in Cadriano, Italy, in the spring of 2018. Approximately 200 seeds of each F_2_ population were sown in rows with an interspacing of 15 cm and an inter‐row distance of 1 m. The F_1_ plants from TM599 × BW630, TM185 × TM599, and TM599 × 1000 were grown in the greenhouse in a peat and vermiculite growing medium (Vigorplant Irish and Baltic peat‐based professional mix) in 15 by 15 by 30 cm polyethylene pots with a day temperature of 22 °C (16 h) and a night temperature of 18 °C (8 h). Greenhouse lighting was a mix of natural light supplemented with artificial light by 400 W high‐pressure sodium lamps (Sylvania SHP‐TS 400W Grolux).

### 2.2 Phenotyping of the F_2_ populations

To determine the segregation of the TM599 and TM4118 necrotic traits, the number of mutated and wild‐type plants were counted at flowering time in the field trial. To calculate the percentage of necrotic leaf area, the third leaf from the top of each necrotic F_2_ plant and six parental plants was collected, scanned with a Canon LiDE120 scanner, and the images were analyzed with the software LNC‐Leaf Necrosis Classifier (https://lnc.proteomics.ceitec.cz/home). The vigor index was calculated as the ratio between the number of culms and the plant height. Culms were counted if taller than 40 cm, including awns. Plant height was measured on the tallest culm, including awns.

### 2.3 Whole‐genome shotgun sequencing and variant calling

Lines TM185, TM599, TM1000, BW630, and barley cultivar Villa were WGS sequenced. The DNA was extracted from leaf samples using a commercial kit (Nucleospin Plant II, Macherey‐Nagel). The DNA was sequenced with Illumina HiSeq PE150. Reads were trimmed using TrimmomaticPE v0.39 (Bolger et al., 2014). Reads were aligned to the third version of barley cultivar Morex genome (Mascher et al., 2021) with BWA 0.7.12‐r1039 (Li & Durbin, 2009) and variants in the genomic space were called with GATK 4.2.0.0 (McKenna et al., 2010), filtering for a minimum read depth of 9×, minimum PHRED quality of 40, and minimum quality normalized on depth of 20.

In this work, mutant gene density (MGD, see Results) is defined as the probability that a gene is affected by moderate (missense or missense and splice region variant) or strong (stop gained, stop lost, start lost, splice acceptor, or spice donor variant) mutations, as predicted by SNPEff (Cingolani et al., 2012) v5.0d, across the genome, considering a total number of protein coding genes of 81,687 as in Morex v3 (Mascher et al., 2021).

### 2.4 Bulked segregant analysis and fine mapping

Bulked segregant analysis (Michelmore et al., 1991) was carried out to map the orange spotted DLM mutant phenotype showed by TM599. An F_2_ population obtained from the cross TM599 × Barke was grown in the field. Plants were visually inspected at flowering time, when 15 *nec3* plants and 15 wild‐type plants were selected. Their leaf DNA was individually extracted with the Macherey‐Nagel Nucleospin Plant II kit and added to two bulks in the same quantity for each single plant, reaching a final concentration of 50 ng μl^−1^ for both bulks. The bulks were genotyped with the 9k Illumina Infinium iSelect barley SNP array (Comadran et al., 2012) by TraitGenetics GmbH. The analysis of SNP signal was carried out with GenomeStudio (Illumina), using the theta value as in Hyten et al. (2008) and calculating delta theta as the squared difference between the wild‐type bulk theta and the necrotic bulk theta. To reduce the candidate interval, a Kompetitive allele specific polymerase chain reaction (KASP) analysis was performed using 47 markers on individual DNAs from the same F_2_ population and the cultivars Barke and Morex.

### 2.5 PCR amplification and Sanger sequencing

Primers were designed with the aid of the online tools Primer3 v4.1.0 (Untergasser et al., 2012) and Benchling (www.benchling.com) for the purpose of obtaining two amplicons of ∼760 bp (Supplemental Table S4). We used the Phusion High‐fidelity PCR kit (Thermo Fischer Scientific, Waltham, Inc.) and ran 35 cycles with a denaturation temperature of 98 °C for 5 s, an annealing temperature of 66 °C for 20 s, and an extension temperature of 72 °C for 16 s. The resulting DNA was sequenced with the Sanger method.

### 2.6 Homology modelling calculations

Template search was performed using the HHsearch method implemented in the HHpred server (Söding et al., 2005). The server performs up to eight iterative PSI‐BLAST (Altschul et al., 1997) searches through filtered versions of the nonredundant database from the NCBI. Using the final target alignment, a profile hidden Markov model (Eddy, 1998; Krogh et al., 1994) is calculated. Homologous templates are identified by searching through a weekly updated database containing hidden Markov model for a representative subset of protein data bank sequences. Finally, HHsearch ranks database matches by the probability of the match to be homologous to the target sequence. This is useful to distinguish homologous from nonhomologous matches. HHpred identified ferruginol synthase from Chinese salvia (*Salvia miltiorrhiza* Bunge) as the best template structure for NEC3. For this protein, a high‐resolution X‐ray structure is available (PDB ID 5YLW, resolution 1.70 Å) (https://www.rcsb.org/structure/5ylw) and the sequence identity with NEC3 (29%) allows the generation of a model through homology modelling. The target and template sequences were realigned using the Promals3D server (Pei et al., 2008). The obtained alignment was then used to calculate 100 models of NEC3 using the Modeller v9.21 software (Šali & Blundell, 1993). The heme group solved in the template structure, as well as a water molecule completing the iron ion coordination sphere, was included in the modelling. The best model was selected using the DOPE potential function included in Modeller (Shen & Sali, 2006). A loop optimization routine was used to refine the regions that showed higher than average energy as calculated using the DOPE potential function. The stereochemical quality of the model structure was established using ProCheck (Laskowski et al., 1993). The results of this analysis confirm the reliability of the model structure. The analysis on the model structure, as well as the molecular graphics reported in this article, was performed by using University of California–San Francisco Chimera (Pettersen et al., 2004).

### 2.7 Phylogenetic analysis of *cytochrome P450 CYP71P1* gene family

A collection of 123 sequenced angiosperm species characterized by Zhao et al. (2020) was analyzed. Genomes were downloaded and queried using BLAST+ (v2.11.0) with default parameters using barley CYP41P1 as query. Multiple sequence alignment was performed using kalign (v3.3) using default parameters. To reduce the alignment to informative sites, the Python script QKphylogeny_alignment_analysis.py (https://github.com/matthewmoscou/QKphylogeny/) was used to extract residues in the alignment that were present in at least 20% of species. Phylogenetic analysis was performed with iqtree v1.6.12 (Nguyen et al., 2015). Model selection identified JTT+I+G4 as the appropriate best fit model using Bayesian Information Criterion. Bootstrapping was performed using ultrafast bootstrapping with a total of 1,000 bootstraps. The phylogenetic tree was visualized using iTOL (https://itol.embl.de/).

## 3 RESULTS

### 3.1 Collection of leaf disease lesion mimic mutants in barley

**FIGURE 1:**
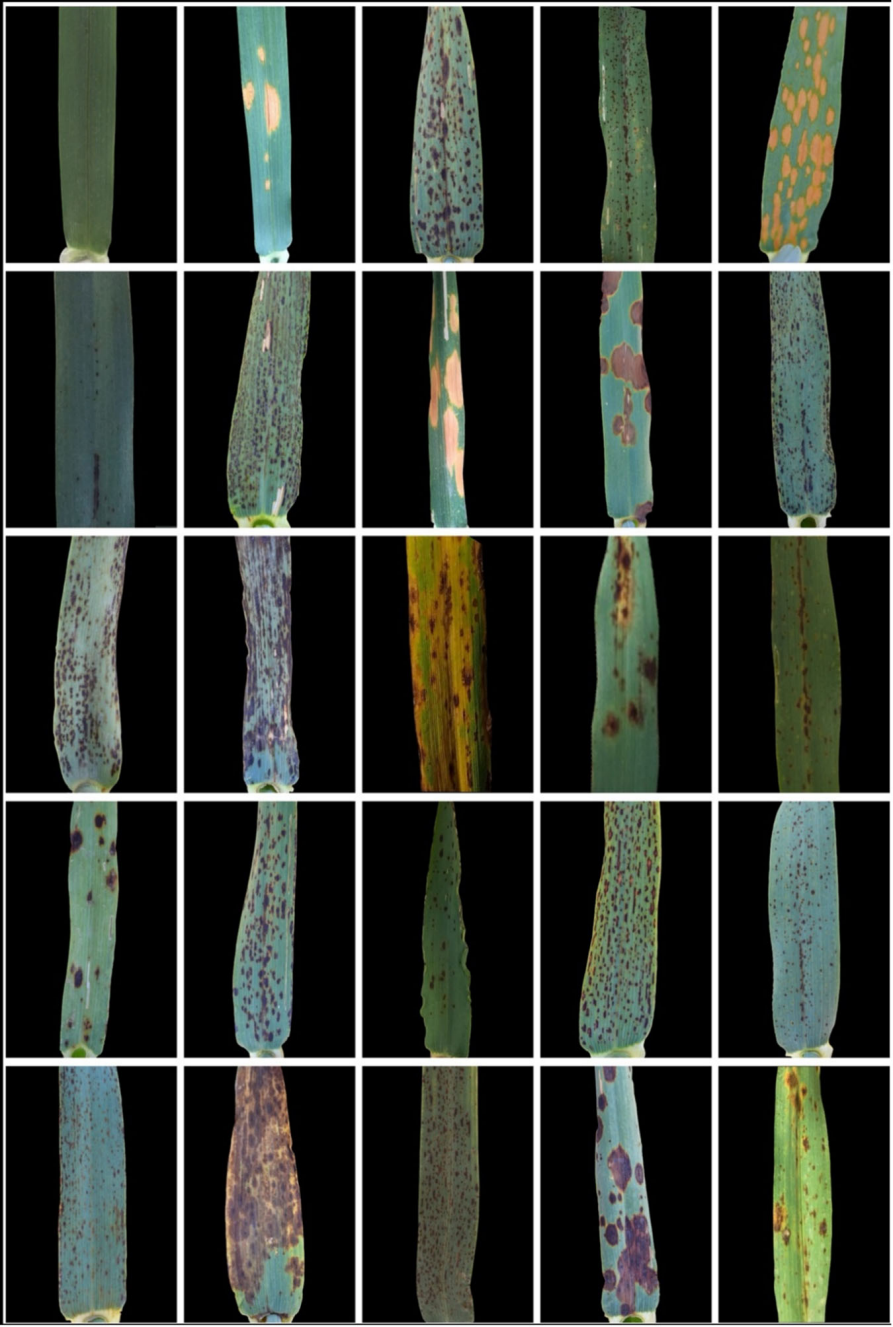
Representative images of the TILLMore disease lesion mimic (DLM) mutant collection. Plants were grown in the field and in the greenhouse, and representative leaves were photographed at flowering time (Zadoks growth stage 6). First row from the top, from left to right: ‘Morex’ (wild type), TM185, TM361, TM588, and TM599; second row: TM849, TM904, TM1000, TM1120, and TM1326; third row: TM1403, TM1708, TM1797, TM1847, and TM2095; fourth row: TM2140, TM2268, TM2286, TM2290, and TM2424; fifth row: TM2695, TM3684, TM4118, TM5776, and TM5866

The chemically mutagenized TM population (3,800 M_5_‐M_6_ lines) was screened in the field in two different years (2016 and 2019) by visual observation for leaf necrosis at the adult stage (from stem elongation to heading) and 34 lines were identified showing stable leaf DLM phenotypes. Necrosis varied in shape, dimension, color, margins, and localization on the leaf blade (Figure 1; Supplemental Table S5). We classified mutant lines into three main categories based on necrosis size: (a) spots, (b) blotches, or (c) extended necrosis on leaf blade. The spots category includes 19 mutant lines with small, regular‐size necrosis, as in TM588 shown in Figure 1. In the following, underlined IDs corresponds to mutant lines shown in Figure 1. Among the spotted lines, four showed lesions that concentrate mainly on the leaf mid vein (TM588, TM849, TM2095, and TM2424), three lines showed lesions that tend to be grouped in short segments parallel to the main leaf vein (TM537, TM2290, and TM2508), 12 lines showed scattered lesions across the leaf blade (TM361, TM904, TM1326, TM1403, TM1428, TM2268, TM2286, TM2695, TM3033, TM3474, TM4118, and TM5617). The blotches category included 10 lines showing medium‐to‐large necrotic blotches on leaf blade, as in TM1120 shown in Figure 1. Three mutants showed orange blotches (TM185, TM599, and TM1000), three burnt orange to light brown blotches (TM1120, TM5721, and TM5866), and four mutants brown to dark brown blotches (TM1674, TM1847, TM2140, and TM5776). When blotch margin was considered, four lines (TM1674, TM1847, TM2140, and TM5866) showed blurred margins, while the remaining six showed net margins. Five lines (TM1120, TM1674, TM2140, TM5721, and TM5866) showed lesions that appeared encircled in a yellow halo. The extended‐necrosis category included five mutants (TM327, TM982, TM1708, TM1797, and TM3684).

### 3.2 Mendelian inheritance and BSA‐based mapping of necrotic mutants

**FIGURE 2:**
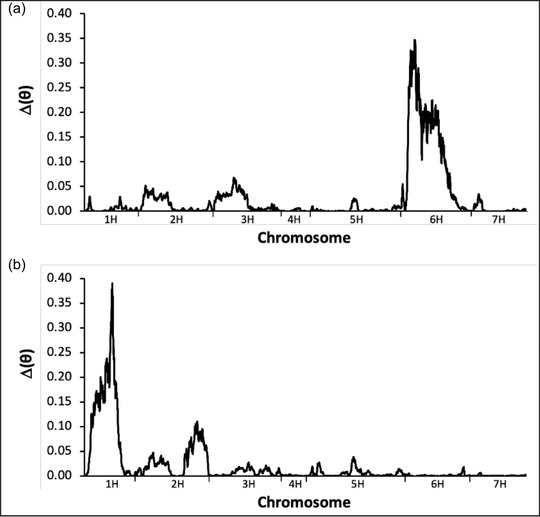
Results of genetic mapping by bulk segregant analysis with high‐density single‐nucleotide polymorphism (SNP) array of disease lesion mimic (DLM) mutants (a) TM599 and (b) TM4118. The *y* axis Δθ (delta theta) value represents allele frequency deviation from null (50:50) at each SNP marker derived following Hyten et al. (2008) with modification (see Materials and Methods section)

Two mutants (TM599, orange blotches) and TM4118 (brown spots) were tested for Mendelian inheritance by outcrossing to a different barley cultivar (Barke), selfing F_1_ plants and growing and phenotyping F_2_ populations. In both cases, necrotic wild‐type plants adhered to the expected ratio of 1:3 for the segregation of a recessive gene (χ^2^ = 0.40, *p* = .52 and χ^2^ = 0.17, *p* = .68, respectively; Supplemental Table S6). In order to genetically map the causative loci, BSA using a high‐density SNP array was performed on F_2_ plants from both populations. The locus for TM599 was mapped on chromosome 6H between markers BOPA2_12_30697 and BOPA2_12_10803 (at ∼21.5 and ∼404.0 Mb, respectively, on Morex ref.3. Figure 2a). The locus for TM4118 was mapped on chromosome 1H between markers BOPA1_ConsensusGBS0342‐1 and SCRI_RS_188218 (∼83.3 and ∼478.8 Mb, respectively, on Morex ref.3. Figure 2b), and following KASP markers development, to an interval of ∼13 Mb encompassing 154 genes. Additional investigations (Supplemental ParagraphS1) showed that TM4118 corresponds to a new allele of the previously cloned *Nec1* (Rostoks et al., 2006). These results indicate that our DLM mutant collection includes mutants under a simple genetic control amenable to gene cloning.

### 3.3 Cloning orange blotch mutations by complementation by sequencing

**FIGURE 3:**
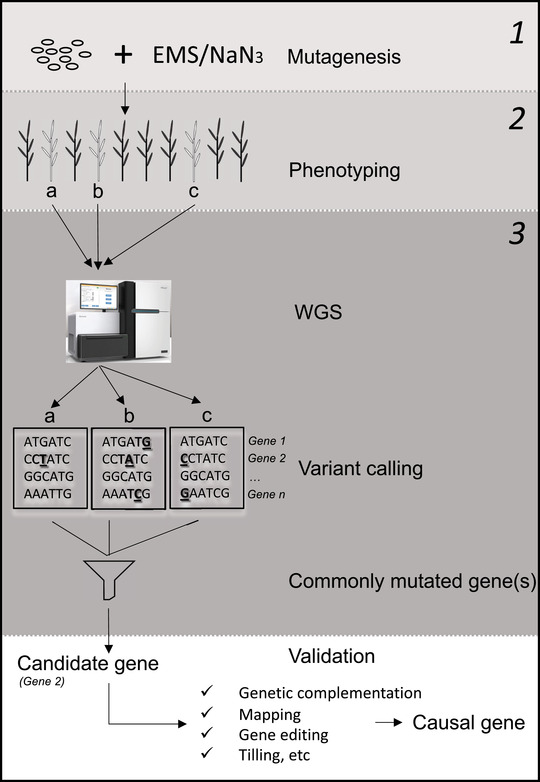
A schematic overview of the complementation‐by‐sequencing (CBS) method that was used to identify the candidate gene responsible for the orange blotch disease lesion mimic (DLM) mutations and the *nec3* locus. (1) Production of a chemically mutagenized population; (2) Identification of mutant plants/lines sharing the same phenotype; (3) WGS sequencing and variant calling in selected plants/lines and identification of commonly mutated genes (*Gene 2*, in the shown example)

We cloned the gene responsible for the orange‐blotched *nec3* phenotype starting from a genomic‐based approach CBS summarized in Figure 3. This approach relies on the feasibility to obtain all the induced mutations in a chemically mutagenized line and the low probability that different mutagenized lines share the same mutated genes. Operatively, CBS includes three steps: (1) a chemically mutagenized population is produced, (2) mutant lines sharing the same target phenotype are identified, and (3) selected mutant lines undergo WGS sequencing, and SNPs are identified by comparison with the genomic sequence of the wild‐type (background) line. A gene mutated in all the selected mutant lines will be considered as candidate given the low probability that this will happen by chance. For example, mutant gene density (probability that a gene is affected by a moderate or strong mutation, that is, a mutation that impacts a splice site, creates a nonsynonymous mutation, frameshift, or early stop codon) can be estimated (Supplemental ParagraphS3). For a mutant gene density of 0.01 in a chemically mutagenized line, the probability that two lines share independent mutations in any of their genes is 0.01 × 0.01 × 30,000 = 3 (with 30,000 = approximate number of genes in a plant diploid genome) or 0.03 in case of three independent lines.

**FIGURE 4:**
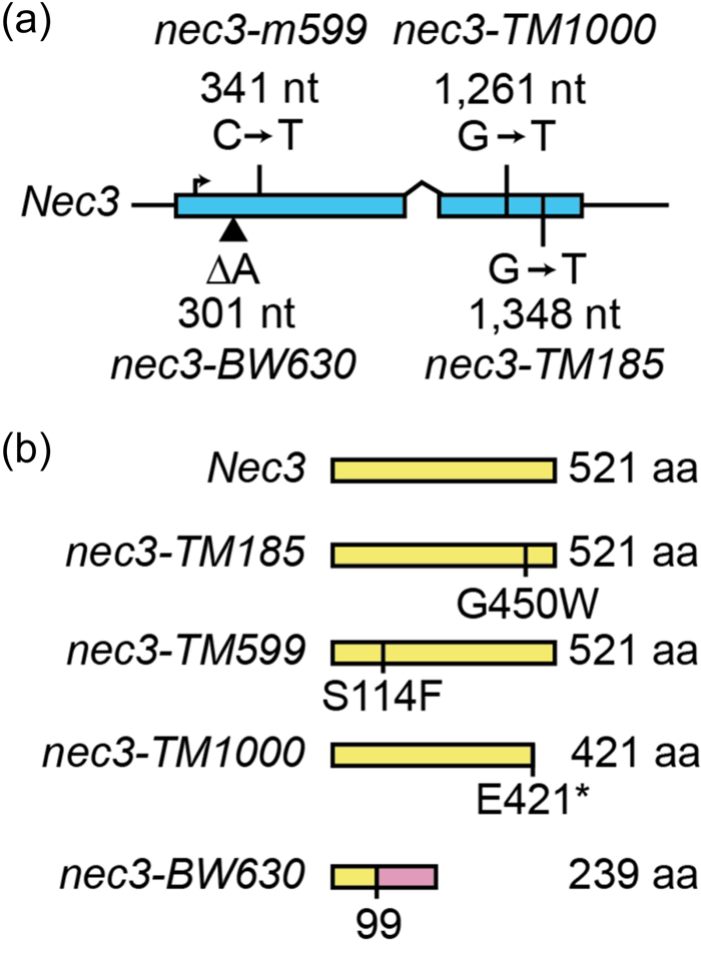
Gene model of *NEC3* (*HORVU.MOREX.r3.6HG0554760.1*) annotated with the induced mutations which were identified in lines BW630, TM185, TM599, and TM1000. (a) Mutations at DNA sequence level. (b) Mutations at amino acid sequence level. Additional details are provided in Supplemental Figure S1

Given the Mendelian inheritance of the orange spot nec3‐like phenotype showed by TM599, and the availability of two additional lines showing the same phenotype [TM185 and TM1000 from the same TM population (Figure 1)], we applied CBS to clone the underlying gene. Whole‐genome shotgun sequencing and SNP extraction resulted in a total of 375, 234, and 78 functional SNPs in TM185, TM599, and TM1000, respectively, (Table 1) corresponding to a number of functionally mutated genes of 369, 234, and 77 and to a mutant gene density of 0.005, 0.003, and 0.001, respectively (average mutant gene density = 0.003). After comparison, the three lines were shown to share only one mutated gene on chromosome 6H (Table 2; Supplemental Figure S1; Figure 4), gene model *HORVU.MOREX.r3.6HG0554760*, annotated as encoding a cytochrome P450 family protein. Table 2 lists the induced SNPs on the gene found in the three mutants. TM185 has the substitution of guanine with thymine at position 1,384 compared with Morex, it causes a missense moderate mutation and an amino acid replacement of glycine at position 450 with tryptophan. TM599 has the substitution of cytosine with thymine at position 341 that causes a moderate mutation and the replacement of serine at position 114 with a phenylalanine. TM1000 has the substitution of guanine with thymine at position 1261 that causes a stop gained strong mutation and the replacement of glutamic acid at 421 with a stop codon. These mutations were confirmed by target Sanger sequencing (Supplemental Figure S2). Notably, this gene lies in the same 6H chromosome region highlighted by BSA.

**TABLE 1:** Mutation discovery by whole‐genome shotgun sequencing in the three orange blotch disease lesion mimic (DLM) mutants identified within the chemically mutagenized population TILLMore

**Mutant**	**SNP density^a^**	**Functional^b^**	**High effect^c^**	**Moderate effect^d^**	**TS^e^**	**TS^f^**
	**kb**	**No**.				**%**
TM185	108.2	375	21	354	32,248	95.23
TM599	182.2	234	19	215	21,842	94.17
TM1000	310.1	78	5	73	6,917	89.01

^a^SNP, single‐nucleotide polymorphism. Length of genomic region per SNP (Supplemental Paragraph S2).

^b^Includes high and moderate effect mutations on genes.

^c^Includes stop gained, stop lost, start lost, splice acceptor, or spice donor variant.

^d^Includes missense variant or missense and splice region variant. Both (c) and (d) according to SNPEff (see Materials and Methods section).

^e^Number of transitions.

^f^Percentage of transitions on the total number of mutations in (a).

**TABLE 2:** Induced mutations identified in *HORVU.MOREX.r3.6HG0554760* (*NEC3*) in the three orange blotch disease lesion mimic (DLM) mutants

**Line**	**Chromosome**	**Position**	**REF**	**ALT**	**Q**	**DP**	**AD**	**Variant**	**Magnitude**	**SEQ**	**PROT**
TM185	6	41,417,210	G	T	659	17	0/17	Missense	Moderate	c.1348G > T	p.Gly450Trp
TM599	6	41,416,044	C	T	841	22	0/22	Missense	Moderate	c.341C > T	p.Ser114Phe
TM1000	6	41,417,123	G	T	890	21	0/21	Stop gain	High	c.1261G > T	p.421Glu*

*Note*. REF, reference base; ALT, alternative base; Q, quality—Phred‐scaled quality score for the assertion made in ALT, that is, *–*10log10 prob(call in ALT is wrong); DP, read depth at this position; AD, allelic depths for the REF and ALT alleles in the order listed; Variant, protein‐coding gene transcript effect predictions according to SNPEff: missense = change of one or more bases, resulting in a different amino acid sequence, transcript length is preserved. stop_gain = at least one base of a codon is changed, resulting in a premature stop codon, leading to a shortened transcript. Magnitude, putative variant impact according to SNPEff (see Materials and Methods section); SEQ, relative position inside the gene and sequence of the mutation; PROT, position and sequence of the mutation on the protein.

### 3.4 Functional validation of *cytochrome P450* as causal gene for the orange blotch and the historical *nec3* barley mutations

Functional validation of *cytochrome P450* was obtained following three approaches. Firstly, we fine mapped the causal DLM locus in TM599 by developing and mapping KASP markers down to a region of ∼165 kb interval on chromosome 6H, between markers 1_0355_120_R and morex_contig_6964_765_F, at 41,293,242 and 41,458,908 bp, respectively, which included six genes. Among these genes, only *HORVU.MOREX.r3.6HG0554760* showed a mutation in TM599 (the C > T substitution at position 41,416,044 bp that was highlighted above). Second, complementation tests were carried out by producing all the crosses between the three orange blotched DLM lines. All the F_1_ plants displayed the typical orange necrotic phenotype (indicating no complementation), thus suggesting that the three lines share the same causal gene (Supplemental Figure S3). Third, we carried out a complementation test between TM599 and BW630, which carries an historical *nec3* mutant allele that exhibits a similar orange spot DLM phenotype to TM185, TM599, and TM1000 (Druka et al., 2011). Also in this case, no complementation was observed in the F_1_ plants, suggesting that BW630 shares the same causal gene as our three DLM mutants. Lastly, we searched for mutations in *cytochrome P450* in BW630, which was shown to carry a deletion of an adenine that causes an altered open reading frame of 242 amino acids in *nec3* (Figure 4) as compared with its wild‐type sequence (from Villa), in agreement with the X‐ray‐based random mutagenesis that was used (Häuser & Fischbeck, 1976). Taken together, these results validate *HORVU.MOREX.r3.6HG0554760*, a predicted cytochrome P450 encoding gene, as the causal gene responsible for the orange blotch DLM mutations and the historical *nec3* barley locus. In turn, these results validate the CBS approach for identifying candidate genes in chemically mutagenized populations.

### 3.5 Homology modelling of *nec3*

**FIGURE 5:**
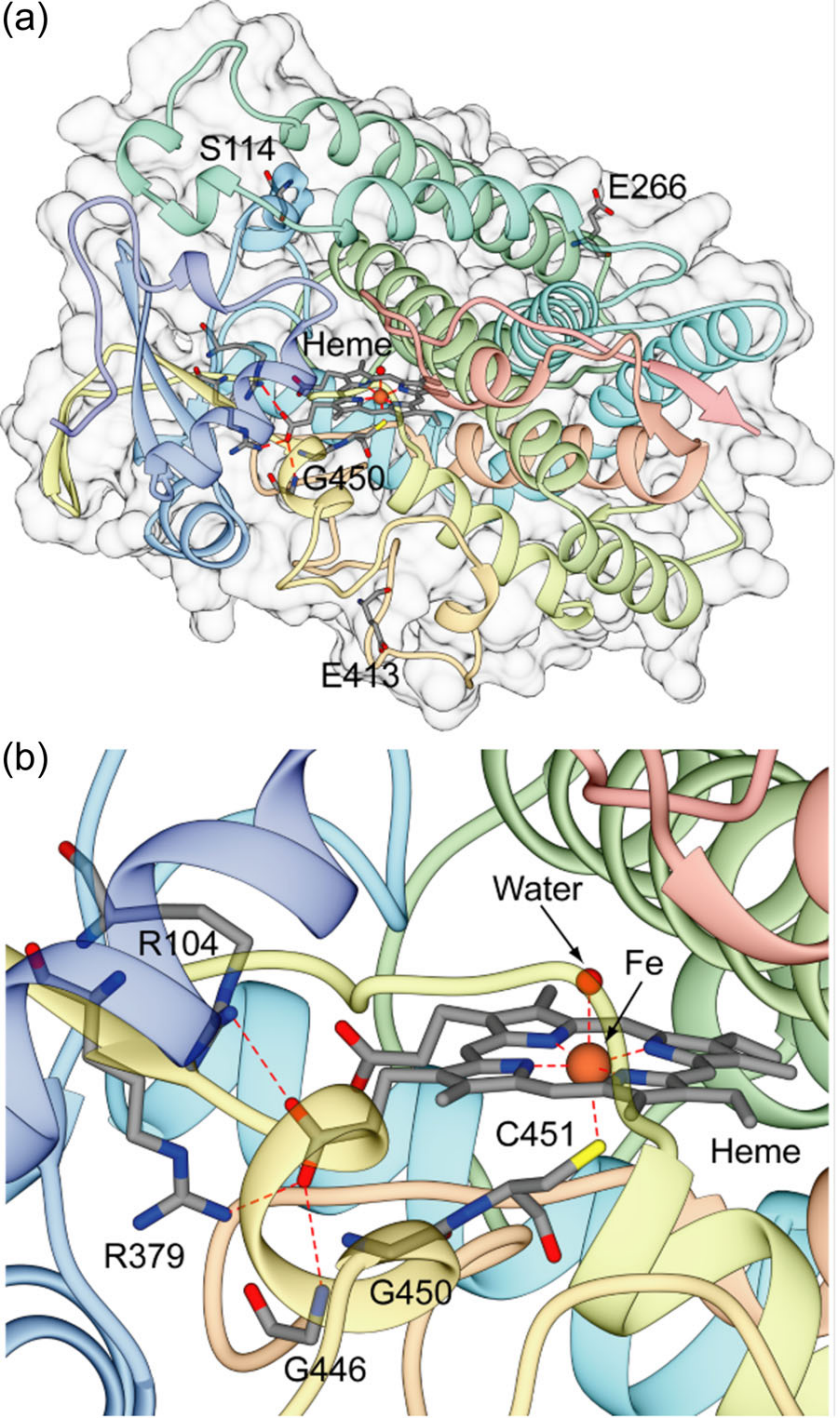
NEC3 model structure. (a) Ribbon diagram and molecular surface of the NEC3 model structure. The ribbons are colored from blue to red by going from the N‐ to the C‐terminal. The heme group and the residues cited in the text are reported as sticks colored according to the atom type. (b) Detail of the heme group and of the residues coordinating the iron ion or H‐bonding the heme group

The structural determinants associated with mutations in *nec3* were analyzed through structural modelling of the protein. The NEC3 protein model structure has the typical tertiary structure of globular proteins and is composed almost entirely by α‐helices and by three small antiparallel β‐sheets (Figure 5). The heme group is hosted in a large cavity in the center of the protein and is held in place through the formation of a coordination bond between the side chain of Cys451 and the central iron ion. The iron ion is also bound to a water molecule completing a slightly distorted octahedral coordination geometry of the metal. The heme group is also H‐bonded to Arg104, Arg379, and Gly446, further stabilizing the binding pose of the prosthetic group in its pocket. The mutation identified in the TM185 mutant line (G450W) can interfere with the positioning of the heme group. Indeed, Gly450 is located in the heme pocket and a substitution with a larger residue, such as a tryptophan, can impede the correct collocation of the heme group. Moreover, Gly450 is next to the iron‐coordinating residue Cys451 and a mutation at this position can impair the conformation of the loop hosting Cys451 and subsequently change the position of the heme group. In the case of TM599 mutation (S114F), the mutation of Ser114 with an aromatic and hydrophobic residue on the surface of the protein can affect the solubility of the protein or can cause some aggregation processes similarly to the paradigmatic case of hemoglobin in the sickle‐cell disease (Ashley‐Koch et al., 2000). Finally, mutant TM1000 is characterized by a large deletion involving a large portion of the C‐terminal region of the protein (residues 421–507) affecting several structural elements composing the heme‐binding pocket, which can be able to impair the correct positioning of the heme group and possibly the folding of the entire protein.

### 3.6 Cytochrome P450 and its evolution in plants

**FIGURE 6:**
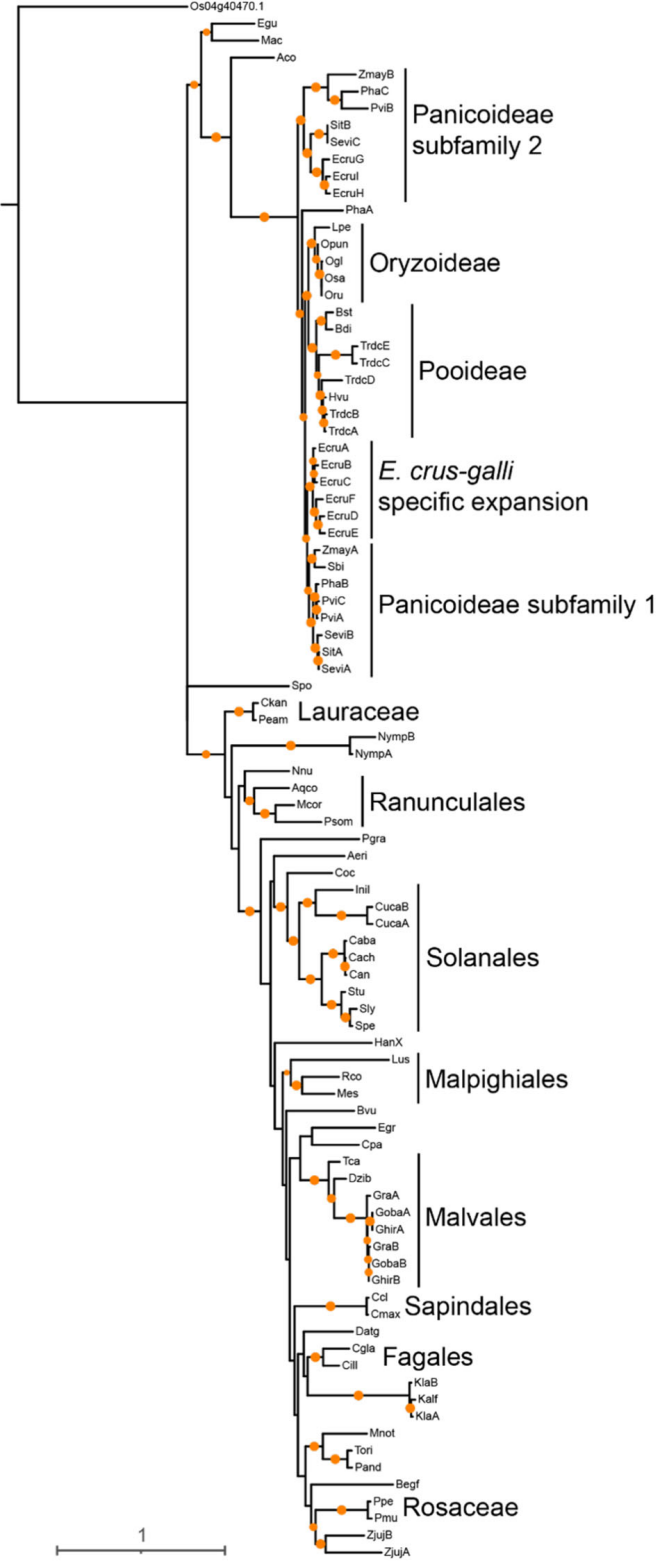
Phylogenetic tree of the cytochrome P450 CYP71P1 protein family. Multiple sequence alignment was performed using MUSCLE (v3.8.31). Phylogenetic tree was constructed using iqtree (v1.6.12). Support is based on 1,000 bootstraps with orange circles indicating at least 80% support. Order and family are annotated when all members are present within a clade with bootstrap support. Rice Os04g40470 was used as outgroup

To identify the evolutionary origin of *NEC3*, we identified cytochrome P450 domain‐containing proteins from barley (400 proteins), rice (409 proteins), and *Brachypodium distachyon* (L.) Beauv. (347 proteins). The maximum likelihood tree using the multiple sequence alignment of cytochrome P450 found that *NEC3* belongs to the *CYP71P1* gene family (Fujiwara et al., 2010). Previous work in rice found that the necrotic lesion mutant *sekiguchi lesion* (*sl*) encodes *CYP71P1*, which catalyzes the conversion of tryptamine to serotonin (Fujiwara et al., 2010). Barley *nec3* mutants phenocopy the rice *sl* mutants with having orange lesions on leaves (Kiyosawa, 1970; Sekiguchi & Furuta, 1965). The presence of *CYP71P1* in rice and barley indicates that this gene family emerged prior to the divergence of these two species. Previous work had found orthologs of *CYP71P1* in wheat, *B. distachyon*, foxtail millet (*Setaria italica* (L.) P. Beauv.), sorghum *[Sorghum bicolor* (L.) Moench], and maize, but absent in *Arabidopsis*, rape (*Brassica napus L*.), soybean, and barrel clover (*Medicago truncatula* Gaertn.) (Lu et al., 2018). To define the boundaries of the presence–absence variation of the *CYP71P1* gene family, we used BLAST to identify putative orthologs of *HvCYP71P1* in angiosperms based on a set of 123 species (52 families; 31 orders) used by Zhao et al. (2020). Phylogenetic analysis of putative orthologs found that alignment identity closely matched presence within the *CYP71P1* gene family (Figure 6). The *CYP71P1* gene family phylogeny largely reflects the species tree of the angiosperms split into monocots and dicots. No evidence was found for the presence of the *CYP71P1* gene family in gymnosperms, ferns, lycophytes, or bryophytes. The most substantial lineage‐specific expansions occur within the monocots including an ancient duplication within the Panicoideae (subfamily 1 and subfamily 2), a duplication of *CYP71P1* from chromosome group 6 to chromosomes 4B and 5A in durum wheat [*Triticum turgidum* L. subsp. *Durum* (Desf.) van Slageren], and a recent expansion in cockspur grass [*Echinochloa crus‐galli* (L.) P. Beauv.]. Several families are absent in the *CYP71P1* gene family phylogeny. Overlay of the presence or absence of the *CYP71P1* gene family found substantial variation throughout angiosperms (Supplemental Figure S5). Evaluation of all families where at least two species are present showed that five families lack *CYP71P1*: Brassicaceae (9 species), Cleomaceae (2 species), Orchidaceae (2 species), Fabaceae (12 species), and Cucurbitaceae (4 species). Taken together, these results show that *CYP71P1* is only found in angiosperms, is widely present in diverse monocot and dicot species, and has been dispensable in some lineages.

The domain structure of CYP71P1 includes a predicted chloroplast localization signal peptide, proline‐rich region, K‐helix, and heme‐binding motifs. We re‐evaluated the mutational landscape in *CYP71P1* family members in rice and barley compared with conservation in amino acid composition in the multiple sequence alignment (Supplemental Figure S6). The barley mutant S114F, identified in TM, is a highly conserved position (86 Ser, 4 Ala, and 1 Phe) with the 4 Ala (ZmayB, PhaC, PviB, TrdcD) and 1 Phe (GraB) in species with multiple *CYP71P1* genes. Rice mutant T314I (CM2229) is highly conserved with 89 Thr and 1 Ala, with the single Ala in PhaA from a member of the gene family in Hall's witchgrass (*Panicum hallii* Vasey). A gap in the position was observed in TrdcD (another duplication from polyploidization and likely a pseudogene). Rice mutant G455D in the heme‐binding motif was invariant in 89 CYP71P1 proteins except for a gap in TrdcD. These results show that all loss‐of‐function mutations that we identified in this study occur in highly conserved sites in *CYP71P1*.

## 4 DISCUSSION

Disease lesion mimic mutants are invaluable tools to dissect the plant defense mechanisms. The collection of 34 DLM barley mutant lines presented in this study significantly integrates existing resources in barley, where ∼30 DLM mutant lines were previously described (Druka et al., 2011; Lundqvist et al., 1997; Rostoks et al., 2003). Once thoroughly characterized, our collection is expected to unveil new loci and new alleles at known loci. In this work, TM4118 was shown to be a new allele of the known DLM gene *nec1* (Rostoks et al., 2006), whereas TM185, TM599, and TM1000 contributed three independent alleles of *nec3* and allowed us to clone the underlying gene. Gene *nec3*, or *necrotic leaf spot 3*, is a historical barley mutation (Häuser & Fischbeck, 1976) for which different induced alleles are available causing tan, orange, or brown necrotic blotches on leaf sheath and blade persisting to maturity (Lundqvist et al., 1997). Some *nec3* alleles were shown to have a negative effect on plant vigor (Druka et al., 2011). By CBS, here we have shown that *NEC3* corresponds to a *cytochrome P450 CYP71P1*. Interestingly, the rice ortholog *Os12g16720*, was found as the causal gene for DLM mutants both in japonica (Cui et al., 2021; Fujiwara et al., 2010) and indica (Tian et al., 2020; Zheng et al., 2021) genetic backgrounds. These works clarified that CYP71P1 has tryptamine5‐hydroxylase enzyme activity and catalyzed the conversion of tryptamine to serotonin (Fujiwara et al., 2010). When CYP71P1 is defective, high endogenous oxidation level leads to cell death occurrence and blotches formation, which is consistent with the role of serotonin as an effective internal reactive oxygen species scavenger (Tian et al., 2020). Other work suggested a potential role of P450 CYP71P1 on insect resistance through its serotonin synthesis effect (Lu et al., 2018) and on chloroplast development or function (Cui et al., 2021). In this work, we have identified three *HvCYP71P1* alleles and confirmed their genetic relationship with the historical mutant *nec3*. Our work shows that *NEC3* is likely conserved in function between rice and barley, which facilitates further work aimed at elucidating its role in response to biotic and abiotic stresses.

Serotonin (5‐hydroxytryptamine) is widely found in animals and plants, playing a role in a diverse range of physiological activities (Akula et al., 2011). The biosynthesis of serotonin in plants occurs through a two‐step process: tryptophan is catalyzed into tryptamine by tryptophan decarboxylase (TDC; EC 4.1.1.28) (Berlin et al., 1993; De Luca et al., 1989), succeeded by catalysis of tryptamine by tryptamine 5‐hydroxylase (T5H) to form serotonin (Schröder et al., 1999). Investigations in the biosynthesis of serotonin in St. John's‐wort (*Hypericum perforatum* L.) identified a similar pathway as mammals involving 5‐hydroxytryptophan (Murch et al., 2000). Using phylogenetic analysis of the *CYP71P1* gene family, we discovered that it is an ancient gene family that emerged early in angiosperm evolution. While *CYP71P1* is found in both monocots and dicots, it has been lost in several lineages. The independent loss of *CYP71P1* in different plant lineages does not appear to be correlated with substantial reduction in serotonin levels (Erland et al., 2016). This supports the hypotheses that at least two pathways exist for serotonin biosynthesis or, alternatively, another enzyme exists with T5H activity. In part, serotonin biosynthesis may occur via TDC, which has both TDC and T5H activity (Park et al., 2008).

We propose CBS as a novel nongenetic, sequencing‐only method to rapidly identify a candidate gene responsible for a mutant phenotype provided the existence of two or more individuals showing the same phenotype and originating from the same mutant collection. CBS relies on the simple rationale that it is possible to identify most or all induced mutations and that individuals sharing the same phenotype should also share the same mutated gene, albeit with different within‐gene mutations. CBS can be applied to any species that can undergo random mutagenesis and is not restricted to a specific pedigree or generational step (although homozygous mutations should be available in order to target recessive mutations). As detailed below, CBS can be applied to most TILLING populations. Additionally, as CBS is not based on meiotic recombination, it can target genes located anywhere in the genome, including highly repetitive, low‐recombinogenic regions, usually not accessible to fine genetic mapping and positional cloning (Taagen et al., 2021).

The CBS approach depends on some assumptions that may limit its applicability. First, theoretically, CBS works best if mutation load is limited, otherwise a pair or a triplet of individuals sharing the same phenotype would also share too many mutations, preventing any candidate gene prioritization. Assessing the effect of mutation load is straightforward by applying the formula SMG = MGD*^n^* × *g*, where SMG is the number of functionally (missense or stronger) mutated genes shared between lines; MGD is the mutant gene density, that is, probability of a gene to carry a functional mutation; *n* is the number of plants showing a given phenotype; and *g* is the number of genes in the genome. The MGD can be derived empirically by sequencing data or by TILLING results (Wang et al., 2012). Chemical mutagenesis in plants was shown to reach an induced SNP density of ∼1/100 kb, beyond which sterility and viability become limiting (Schreiber et al., 2019; Wang et al., 2012). At this SNP density, a homozygous line will carry on average 210 functionally mutated genes (Supplemental Paragraph S3), corresponding to MGD = 0.006 (210/35,000), thus, SMG between two lines will be ∼1, likely allowing to identify the effective candidate gene. For the most common lower induced SNP densities (i.e., ∼1/500 kb) (Wang et al., 2012) the number of mutated genes per line will be in the order of 50 and SMG between two lines will drop to <1, strongly reducing the risk of a false positive candidate gene identification. Second, population sizes should be large enough to warrant the presence of a second or third individual independently mutated in the same gene (this condition known as saturation), given a known mutation density. Estimates of mutant population sizes reaching saturation have been thoroughly worked out and range from few thousands to 10,000 individuals (Wang et al., 2012). Third, CBS, like any other forward‐ or reverse‐genetics approach, will be impacted by the complexity of the genetic architecture underlying a given phenotype (i.e., whenever different mutated genes would cause the same phenotype). While in this situation CBS will not identify a common gene, the increasing knowledge of biochemical metabolic pathways, including their regulation, could at least lead to the identification of the relevant biochemical pathway and gene network where the different mutations act. For this purpose, tools to explore gene knowledge, that is, KnetMine (Hassani‐Pak et al., 2020) and metabolic networks, that is, PMN, Plant Metabolic Pathway Databases (Schläpfer et al., 2017), are available for many model and crop species. A fourth assumption is the availability of a high‐quality reference genome, which is necessary for reliable SNP calling in CBS Step 3. However, the current favorable trend of cost reduction and throughput increase should soon make this point less relevant for most species, as exemplified in barley (Mascher et al., 2021).

## 5 CONCLUSIONS

We showed that CBS can effectively clone a gene responsible for DLM mutations in a chemically mutagenized collection. The target gene was shown to correspond to the historically known barley *nec3* locus and to encode a *cytochrome P450 CYP71P1*. *CYP71P1* is found in most angiosperms, both monocots and dicots, but is lost in some lineages, including *Arabidopsis*. Because CYP71P1 catalyzes the last step in the biosynthesis of serotonin, which affects reactive oxygen species scavenging, chloroplast development, and fungi and insect resistance, this information could contribute to genetically improve barley for enhanced resistance to parasites.

## AUTHOR CONTRIBUTIONS

Serena Rosignoli: Data curation; Formal analysis; Investigation; Methodology; Software; Validation; Visualization; Writing – original draft; Writing – review & editing. Francesco Cosenza: Formal analysis; Investigation; Writing – original draft. Laura Civolani: Formal analysis; Investigation; Validation. Matthew J. Moscou: Conceptualization, Data curation, Formal analysis, Investigation, Methodology, Resources, Software, Supervision, Visualization, Writing–original draft, Writing–review & editing. Francesco Musiani: Conceptualization; Data curation; Formal analysis; Investigation; Methodology; Writing – original draft. Cristian Forestan: Methodology; Validation; Writing – original draft; Writing – review & editing. Sara Giulia Milner: Data curation; Formal analysis; Methodology; Software; Supervision; Visualization; Writing – original draft. Castrense Savojardo: Formal analysis; Methodology; Validation; Writing – review & editing. Roberto Tuberosa: Conceptualization; Funding acquisition; Methodology; Resources; Writing – review & editing. Silvio Salvi: Conceptualization; Data curation; Formal analysis; Funding acquisition; Investigation; Methodology; Project administration; Resources; Supervision; Validation; Visualization; Writing – original draft; Writing – review & editing.

## CONFLICT OF INTEREST

The authors declare that there are no conflicts of interest.

## Supporting information

(files = Supplemental.docx; WGSDATA_TM185.csv, WGSDATA_TM599.csv, WGSDATA_TM1000.csv, WGSDATA_TM4118.csv)
**Supplemental Paragraph S1**. TM4118 has the same causal gene as the previously described *nec1* mutant.
**Supplemental Paragraph S2**. Calculations and rationale for mutations filtering of Table 1.
**Supplemental Paragraph S3**. Estimation of mutant gene density (MGD) in a genome.
**Supplemental Table S1**. Chromosome length for Morex v3 reference genome.
**Supplemental Table S2**. Number of SNPs and mutation density (genomic region length/number of SNPs) in kilobases, for each chromosome, of the mutants TM185, TM599, and TM1000.
**Supplemental Table S3**. Values involved in mutation density (Table 1) calculation.
**Supplemental Table S4**. PCR primers used in the validation of mutant alleles of *nec3*.
**Supplemental Table S5**. TILLMore necrotic collection.
**Supplemental Table S6**. Mendelian inheritance of necrotic mutations carried by TM599 (large orange blotches) and TM4118 (small brown spots).
**Supplemental Table S7**. Model structure evaluation. Results of the Procheck and Prosa analysis done on the NEC3 model structure and on the ferruginol synthase from *Salvia miltiorrhiza* (Chinese sage) structure (PDB id 5YLW).
**Supplemental Figure S1**. Multialignment of *nec3* alleles. Alignment of the a) coding sequences and b) proteic sequences of the *nec3* gene from TM185, TM599 and TM1000 to the reference sequence (Morex, wild type). The layout was generated with Jalview v.2.11 (Waterhouse et al., 2009). TM1000 has three SNPs compared to Morex, only the third one (c.1261G > T) causing a STOP codon has been caused by mutagenesis whilst the other two, causing missense variants, were previously present, derived from natural heterogeneity.
**Supplemental Figure S2**. Sanger sequencing results of the *NEC3* alleles. On the left column: chromatograms of sequences from mutant plants in the region of previously identified SNPs. In red circle: SNPs mutations. On the right column: chromatograms of sequences from wild‐type Morex plants in the same region as on the left. The green circle highlights the reference base corresponding to mutations in the red circle.
**Supplemental Figure S3**. Results of complementation test between *nec3* mutants. From left to right: F_1_ plants for TM599 × BW630, TM599 × TM1000, TM185 × TM599. All F_1_ plants from the three crosses between the four DLM *nec3*‐like mutants showed the distinctive orange blotch phenotype.
**Supplemental Figure S4**. Sequence alignment of NEC3 and the template structure. PROMALS3D sequence alignment with the ferruginol synthase from *Salvia miltiorrhiza* (Chinese sage) sequence (5YLW_A). Residues in red and italics were not modelled due to the absence of available structural template structures. Promals3D consensus amino acid sequence symbols are also shown: conserved amino acids are in uppercase letters; aliphatic (I, V, L): l; aromatic (Y, H, W, F): @; hydrophobic (W, F, Y, M, L, I, V, A, C, T, H): h; alcohol (S, T): o; polar residues (D, E, H, K, N, Q, R, S, T): p; tiny (A, G, C, S): t; small (A, G, C, S, V, N, D, T, P): s; bulky residues (E, F, I, K, L, M, Q, R, W, Y): b; positively charged (K, R, H): +; negatively charged (D, E): ‐; charged (D, E, K, R, H): c.
**Supplemental Figure S5**. Substantial presence/absence variation in the *CYP71P1* gene family throughout angiosperms. Presence (orange) or absence (blue) is based on iterative BLAST analysis to identify putative *CYP71P1* homologs and phylogenetic analysis to confirm orthology. Grey indicates that the genome was inaccessible. Angiosperm phylogenetic tree is from Zhao et al. (2020).
**Supplemental Figure S6**. Mutational landscape of the *CYP71P1* gene family. CYP71P1 carries a predicted chloroplast localization signal peptide, proline‐rich region, K‐helix, and heme‐binding motifs. Barley mutant S114F is a highly conserved position (86 Ser, 4 Ala, and 1 Phe) with the 4 Ala (ZmayB, PhaC, PviB, TrdcD) and 1 Phe (GraB) in species with multiple *CYP71P1* genes. Rice mutant T314I (CM2229) is highly conserved with 89 Thr and 1 Ala, with the single Ala in PhaA from a member of the gene family in *P. hallii*). A gap in the position was observed in TrdcD (another duplication from polyploidization and likely a pseudogene). Rice mutant G455D in the heme‐binding motif was invariant in 89 CYP71P1 proteins except for a gap in TrdcD.
In WGSDATA_TM185.csv, WGSDATA_TM599.csv, WGSDATA_TM1000.csv, and WGSDATA_TM4118.csv
List of the SNP mutations called in the genome of TM mutants TM185, TM599, TM1000 and TM4118 after their WGS, in comparison to barley cv. Morex v.3.
