# Supplementary material for: Cloning the barley nec3 disease lesion mimic mutant using complementation by sequencing
Source: Plant Genome. 2022 Mar 18;15(2):e20187. doi: 10.1002/tpg2.20187 (PMC12807410; doi:10.1002/tpg2.20187)
Supplement: Supplementary file 1 [file TPG2-15-e20187-s001.docx]

**SUPPLEMENTAL MATERIAL FOR:**

**CLONING THE BARLEY NEC3 DISEASE LESION MUTANT USING COMPLEMENTATION BY SEQUENCING**

Serena Rosignoli, Francesco Cosenza, Matthew J. Moscou, Laura Civolani, Francesco Musiani, Cristian Forestan, Sara Giulia Milner, Castrense Savojardo, Roberto Tuberosa, Silvio Salvi

**Number of pages (including this one):** 23.

**Number of supplemental paragraphs:** 3.

**Number of supplemental figures:** 6.

**Number of supplemental tables:** 7.

**Supplemental Paragraph S1. TM4118 has the same causal gene as the previously described *nec1* mutant**

TM4118 displays the same necrotic brown spots of the *nec1* barley mutant which has previously been described and cloned (Rostoks et al., 2006). The F_2_ from TM4118 × Barke demonstrates the mendelian recessive inheritance of *nec1-like* (χ^2^ = 0.14, n = 162). Bulked segregant analysis on the same F_2_ population followed by fine mapping with KASP markers identified an interval of 5.3 Mb on chromosome 1H encompassing a region of 469 kb. Following the development and analysis of 16 KASP markers, the causal locus was mapped between markers SCRI_RS_157039 (62.8 cM) and SCRI_RS_188360 (70.3 cM). The region between these two markers includes a physical range of 13,190,391 bp, within which 154 high confidence genes are contained. This region corresponds with the previously mapped and cloned DLM *Nec1* locus (Rostoks et al., 2006), encoding a cyclic nucleotide-gated channel. We proceeded with WGS sequencing of TM4118 with Illumina HiSeq PE150. A total of 7,549 of SNPs were mapped. The estimated mutation load on the wholeTM4118 genome is 11280.1, equivalent to one mutation every 443 kb (very close to the mean mutation density estimated after TILLING experiments in TILLMore, 1/350 kb. Talamè et al. 2008). Only one SNP was mapped inside the TM4118 target physical interval found by fine mapping: a G>A substitution at position 1H: 438,323,046 which, based on current barley reference genome annotation, causes a stop-gain mutation on *Nec1* (gene model *HORVU.MOREX.r3.1HG0067420*). Therefore, we conclude that TM4118 carries a novel *nec1* mutant allele.

**Supplemental Paragraph S2. Calculations and rationale for mutations filtering of Table 1**

Comparing the number of variants per chromosome (Supplemental Table S2), it was evident that the initial seed stock used for mutagenesis contained plural contaminations from backgrounds that are different from Morex. In particular, TM185 appears to be contaminated on chromosome 6H and TM1000 on chromosomes 3H, and likely on 6H and 7H. This did not represent a limitation to the finding of the candidate gene, but we decided to leave those chromosomes out when determining the TS and TV percentages, otherwise the number of induced mutations would have been outnumbered by the pre-existent natural variants. Details on transitions number and percentage, and mutation density, are reported in Supplemental Table S1 and Supplemental Table S3.

**Supplemental Paragraph S3. Estimation of mutant gene density (MGD) in a genome.**

The maximum SNP density obtainable by chemical mutagenesis in a diploid species is likely around 1 SNP/100 kb (Schreiber et al., 2019; Wang et al., 2012). SNP density can be derived empirically using sequencing data and comparison with wild type reference, or by TILLING. The average coding space of a plant gene was estimated as 1,200 bp (c. 400 aa. Ramírez-Sanchez et al., 2016). Thus, the probability that a gene is hit by a random SNP (= the probability that a gene is mutated = mutant gene density or MGD) approximates to 1.2 kb/100 kb = 0.012 or ≈ 0.01. This is the maximum MGD that appears to be reachable.

Most often, lower MGD will be reached by lower SNP density. MGD can also be used to estimate the total number of mutated genes in a genome.

**a.**

**b.**

**Supplemental Figure S1. Multialignment of *nec3* alleles.** Alignment of the **a)** coding sequences and **b)** proteic sequences of the *nec3* gene from TM185, TM599 and TM1000 to the reference sequence (Morex, wild-type). The layout was generated with Jalview v.2.11 (Waterhouse et al., 2009). TM1000 has three SNPs compared to Morex, only the third one (c.1261G>T) causing a STOP codon has been caused by mutagenesis whilst the other two, causing missense variants, were previously present, derived from natural heterogeneity (See Supplemental Table S2).

**Supplemental Figure S2. Sanger sequencing results of the *NEC3* alleles.** On the left column: chromatograms of sequences from mutant plants in the region of previously identified SNPs. In red circle: SNPs mutations. On the right column: chromatograms of sequences from wild-type Morex plants in the same region as on the left. In green circle: reference base corresponding to mutations in the red circle.

**Supplemental Figure S3. Results of complementation test between *nec3* mutants.** From left to right: F_1_ plants for TM599 × BW630, TM599 × TM1000, TM185 × TM599. All F_1_ plants from the three crosses between the four DLM *nec3*-like mutants showed the distinctive orange blotch phenotype.

**Supplemental Figure S4. Sequence alignment of NEC3 and the template structure.** PROMALS3D sequence alignment with the ferruginol synthase from *Salvia miltiorrhiza* (Chinese sage) sequence (5YLW_A). Residues in red and italics were not modelled due to the absence of available structural template structures. Promals3D consensus amino acid sequence symbols are also shown: conserved amino acids are in uppercase letters; aliphatic (I, V, L): l; aromatic (Y, H, W, F): @; hydrophobic (W, F, Y, M, L, I, V, A, C, T, H): h; alcohol (S, T): o; polar residues (D, E, H, K, N, Q, R, S, T): p; tiny (A, G, C, S): t; small (A, G, C, S, V, N, D, T, P): s; bulky residues (E, F, I, K, L, M, Q, R, W, Y): b; positively charged (K, R, H): +; negatively charged (D, E): -; charged (D, E, K, R, H): c. 

**Supplemental Figure S5. Substantial presence/absence variation in the *CYP71P1* gene family throughout angiosperms.** Presence (orange) or absence (blue) is based on iterative BLAST analysis to identify putative *CYP71P1* homologs and phylogenetic analysis to confirm orthology. Grey indicates that the genome was inaccessible. Angiosperm phylogenetic tree is from Zhao *et al.* (2020).

**Supplemental Figure S6. Mutational landscape of the *CYP71P1* gene family.** *CYP71P1* carries a predicted chloroplast localization signal peptide, proline-rich region, K-helix, and heme-binding motifs. Barley mutant S114F is a highly conserved position (86 Ser, 4 Ala, and 1 Phe) with the 4 Ala (ZmayB, PhaC, PviB, TrdcD) and 1 Phe (GraB) in species with multiple *CYP71P1* genes. Rice mutant T314I (CM2229) is highly conserved with 89 Thr and 1 Ala, with the single Ala in PhaA from a member of the gene family in *P. hallii*). A gap in the position was observed in TrdcD (another duplication from polyploidization and likely a pseudogene). Rice mutant G455D in the heme-binding motif was invariant in 89 CYP71P1 proteins except for a gap in TrdcD.

**Supplemental Table S1**. Chromosome length for Morex v3 reference genome, as available from (Mascher et al., 2021) .

| CHROMOSOME | LENGTH |
| --- | --- |
| 1H | 516,505,932 |
| 2H | 665,585,731 |
| 3H | 621,516,506 |
| 4H | 610,333,535 |
| 5H | 588,218,686 |
| 6H | 561,794,515 |
| 7H | 632,540,561 |
| Un | 29,110,253 |

**Supplemental Table S2**. Number of SNPs and mutation density (genomic region length/number of SNPs) in kilobases, for each chromosome, of the mutants TM185, TM599 and TM1000.

| MUTANT | CHROMOSOME | SNPs | MUTATION DENSITY (kb/SNP) |
| --- | --- | --- | --- |
| TM185 | 1H | 5,295 | 97.5 |
|  | 2H | 6,212 | 107.1 |
|  | 3H | 5,547 | 112.0 |
|  | 4H | 5,375 | 113.6 |
|  | 5H | 5,656 | 104.0 |
|  | 6H | 304,232 | 1.8 |
|  | 7H | 5,391 | 117.3 |
|  | Un | 387 | 75.2 |
| TM599 | 1H | 2,984 | 173.1 |
|  | 2H | 4,109 | 162.0 |
|  | 3H | 3,351 | 185.5 |
|  | 4H | 2,719 | 224.5 |
|  | 5H | 3,631 | 162.0 |
|  | 6H | 3,068 | 183.1 |
|  | 7H | 3,268 | 193.6 |
|  | Un | 65 | 447.9 |
| TM1000 | 1H | 1,723 | 299.8 |
|  | 2H | 2,022 | 329.2 |
|  | 3H | 32,002 | 19.4 |
|  | 4H | 1,958 | 311.7 |
|  | 5H | 2,022 | 290.9 |
|  | 6H | 4,253 | 132.1 |
|  | 7H | 5,614 | 112.7 |
|  | Un | 46 | 632.8 |

**Supplemental Table S3**. Values involved in mutation density (Table 1) calculation.

| MUTANT | SNPs^(a)^ | EXCLUDED CHROMOSOMES^(b)^ | REMAINING SNPs^(c)^ | REMAINING REGION (bp)^(d)^ | MUTATION DENSITY ON REMAINING REGION (kb)^(e)^ |
| --- | --- | --- | --- | --- | --- |
| TM185 | 338,095 | 6H | 33,863 | 3,663,811,204 | 108.2 |
| TM599 | 23,195 | / | 23,195 | 4,225,605,719 | 182.2 |
| TM1000 | 49,640 | 3H, 6H, 7H | 7,771 | 2,409,754,137 | 310.1 |

(a) Total number of SNPs for each mutant line.
(b) Chromosomes excluded from TS count and percentage because likely containing variants pre-existent to mutagenesis.
(c) Number of SNPs remaining after the exclusion of chromosomes.
(d) Length of the remaining region after the exclusion of chromosomes.
(e) New mutation density, as shown in Table1, calculated as the ratio of remaining region length to remaining SNPs number.

**Supplemental Table S4**. PCR primers used in the validation of mutant alleles of *nec3*.

| ID | SEQUENCE |
| --- | --- |
| TAN_F1 | CCACCTCCACCTGCTGAC |
| TAN_R1 | AGGTTGTCGTCGGTGAGC |
| TAN_F2 | ACAACCTCAAAGCCCTCGT |
| TAN_R2 | GGGTCCTTCTTCCTGGTGG |

**Supplemental Table S5**. TILLMore Necrotic collection. Dimensions are based on the main

axis of the lesion, small: main axis < 3mm; medium: main axis between 3 and 6 mm, included;

large: main axis > 6 mm.

| ID | Shape | Margins | Dimension | Colour | Short description |
| --- | --- | --- | --- | --- | --- |
| TM185 | irregular elliptic to circular blotches | distinct | large | orange | large irregular orange blotches with distinct margins |
| TM327 | large irregular necrosis | distinct | large | brown/beige | small brown spots that coalesce into large beige papery area with brown margins |
| TM361 | small to medium circular and elliptic necrosis | distinct | small | brown | small brown circular and elliptic spots that are sometimes bigger and tend to grow on the mid vein |
| TM537 | spot to linear | distinct | small | brown/dark brown | small dark brown spots coalescent into segment > 1cm parallel to leaf vein and visible from both adaxial and abaxial surface |
| TM588 | small brown spots | distinct | small | brown | small spots that tend to coalesce on mid vein |
| TM599 | irregular elliptic to circular blotches | distinct | large | orange | large irregular orange blotches with distinct margins |
| TM849 | small brown spots | distinct | small | dark brown | very small spots that are rare on the leaf but coalesce on the first half of mid vein |
| TM904 | very irregular | blurred | large | brown/dark brown | small brown spots/short segments forming near leaf lateral margins and leaf mid vein associate into a very irregular brown longitudinal necrosis with varied color intensity and blurred in some parts. |
| TM982 | spots | distinct | small | bronze | small bronze spots develop on blade and sheat and their density increases conferring an overall irregular bronze color to all the surface |
| TM1000 | irregular elliptic to circular blotches | distinct | large | orange | large irregular orange blotches with distinct margins |
| TM1120 | irregular elliptic to circular blotches | distinct | large | brown to burnt orange | large irregular burnt orange blotches having irregular oily margins turning to burnt orange blotches with yellow halo |
| TM1326 | short segments / irregular | distinct | small | dark brown to black | small very dark spots converge into irregular short segments parallel to the vein |
| TM1403 | small to medium spots/ irregular | distinct | small | dark brown to black | small dark brown spots converge into medium irregular shapes |
| TM1428 | small to medium spots | distinct | small | dark brown | small brown spots sometimes converge into short segments especially on leaf margins or enlarge to medium spots |
| TM1674 | small spot to large stripes | blurred | large | brown | small brown spots with yellow halo that transforms into irregular cluster of spots or big longitudinal stripes |
| TM1708 | small to medium ellipses | blurred | medium | dark brown to black | small dark elliptic lesions that enlarge and coalesce into big irregularly shaped black lesions |
| TM1797 | brown blotches | blurred | big | brown to burnt orange | medium to big brown to burnt orange blotches and leaf decoloration |
| TM1847 | brown blotches | blurred | big | brown | few big brown blotches with blurred margins and some smaller lesion |
| TM2095 | small brown spots | distinct | small | brown | small light brown lesion mainly on midvein, coalescing |
| TM2140 | large elliptic to circular necrosis | blurred | large | brown | small brown spots evolve to big elliptic-circular brown spots with orange-yellow halo |
| TM2268 | small brown spots | distinct | small | brown | small very dark spots converge into irregular dark brown short segments or blotches |
| TM2286 | small brown spots | distinct | small | brown | sparse brown spots of varying size, discolored and curled leaf margins |
| TM2290 | small bronze spots/short lines | distinct | small | bronze | small brown spots converge into irregular bronze small blotches or short segments parallel to the vein |
| TM2424 | small brown spots + central vein | distinct | small | brown | small brown spots scattered on the leaf that coalesce in a central line on the mid vein |
| TM2508 | small brown spots | distinct | small | brown | small brown spots that tend to be distributed along the leaf veins. |
| TM2695 | small brown spots | blurred | small | brown | small brown spots that sometimes coalesce in short segments |
| TM3033 | small brown spots | distinct | small | brown | small brown spots sparsely scattered |
| TM3474 | small brown ellipses | distinct | small | brown | small brown spots that evolve into elliptic lesions |
| TM3684 | large brown lesions | blurred | large | brown and light brown | big brown blotches enlarge and coalesce covering almost the entire leaf |
| TM4118 | small dark brown spots | distinct | small | brown to dark brown | small dark brown to elliptic spots |
| TM5617 | small burnt orange spots and blotches | distinct | small | burnt orange | small burnt orange spots sometimes grow to larger irregular lesions |
| TM5721 | irregular elliptic to circular blotches | distinct | large | brown to burnt orange | large irregular burnt orange blotches having irregular oily margins turning to burnt orange blotches with yellow halo |
| TM5776 | large dark brown to black elliptic to circular blotches | distinct | large | dark brown to black | large dark brown to black elliptic to circular blotches with varied shape and dimension within the same leaf |
| TM5866 | medium-large bunt orange lesions | blurred | large | burnt orange | medium to large burnt orange irregularly shaped lesions with yellow halo |

**Supplemental Table S6**. Mendelian inheritance of necrotic mutations carried by TM599 (large orange blotches) and TM4118 (small brown spots).

| ID F_2_ | Wild-type | Mutant | Total | Ratio | χ^2^ | P |
| --- | --- | --- | --- | --- | --- | --- |
| TM599 × Barke | 149 | 43 | 192 | 0.29 | 0.40 | 0.52 |
| TM4118× Barke | 114 | 48 | 162 | 0.42 | 0.17 | 0.68 |

**Supplemental Table S7**. Model structure evaluation. Results of the Procheck and Prosa analysis done on the NEC3 model structure and on the ferruginol synthase from *Salvia miltiorrhiza* (Chinese sage) structure (PDB id 5YLW).

| **Protein** | **Procheck Ramachandran plot** | | | | **Procheck**  **G-factor** | **Prosa**  **Z-score** |
| --- | --- | --- | --- | --- | --- | --- |
|  | **Most favored** | **Additionally allowed** | **Generously allowed** | **Disallowed** |  |  |
| NEC3 | 92.8% | 6.9% | 0.2% | 0.0% | -0.12 | -9.27 |
| 5YLW | 95.1% | 4.1% | 0.0% | 0.0% | -0.23 | -9.79 |

Mascher, M., Wicker, T., Jenkins, J., Plott, C., Lux, T., Koh, C. S., . . . Stein, N. (2021). Long-read sequence assembly: a technical evaluation in barley. *The Plant Cell*. https://doi.org/10.1093/plcell/koab077

Ramírez-Sánchez, O., Pérez-Rodríguez, P., Delaye, L., & Tiessen, A. (2016). Plant proteins are smaller because they are encoded by fewer exons than animal proteins. *Genomics, proteomics & bioinformatics*, *14*(6), 357-370. https://doi.org/10.1016/j.gpb.2016.06.003

Rostoks, N., Schmierer, D., Mudie, S., Drader, T., Brueggeman, R., Caldwell, D. G., . . . Kleinhofs, A. (2006). Barley necrotic locus nec1 encodes the cyclic nucleotide-gated ion channel 4 homologous to the Arabidopsis HLM1. *Mol Genet Genomics*, *275*(2), 159-168. https://doi.org/10.1007/s00438-005-0073-9

Schreiber, M., Barakate, A., Uzrek, N., Macaulay, M., Sourdille, A., Morris, J., . . . Waugh,

R. (2019). A highly mutagenised barley (cv. Golden Promise) TILLING population coupled with strategies for screening-by-sequencing. *Plant methods*, *15*(1), 1-14.

https://doi.org/10.1186/s13007-019-0486-9

Waterhouse, A. M., Procter, J. B., Martin, D. M., Clamp, M., & Barton, G. J. (2009). Jalview Version 2--a multiple sequence alignment editor and analysis workbench. *Bioinformatics*, *25*(9), 1189-1191. https://doi.org/10.1093/bioinformatics/btp033

Zhao, T., Xue, J., Kao, S.-m., Li, Z., Zwaenepoel, A., Schranz, M. E., & Van de Peer, Y. (2020). Novel phylogeny of angiosperms inferred from whole-genome microsynteny analysis. *bioRxiv*. https://doi.org/10.1101/2020.01.15.908376
